# Nursing Informaticians in Spain: Scoping Review and Expert-Validated Gap Analysis

**DOI:** 10.2196/83373

**Published:** 2026-04-27

**Authors:** Adrián Marco-Moyano, Patricia Verdú Rodríguez, Manuel Lillo-Crespo

**Affiliations:** 1 University of Alicante, San Vicente del Raspeig, Alicante, Spain; 2 Faculty of Health Sciences, University of Alicante, Carretera de San Vicente del Raspeig, s/n, San Vicente del Raspeig, Alicante, 03690, Spain, 34 965903400 ext 3518

**Keywords:** nursing informatics, nursing informaticians, enfermeras especialistas en sistemas de información, digital health, competency-based education

## Abstract

**Background:**

The role of nursing informaticians is well-established in countries like the United States, Canada, and Australia, supported by competency frameworks and educational programs that enable nurses to lead technological integration in health care. However, in Spain, this role is not formally recognized, and specialized university training is scarce, creating a significant gap in digital health leadership among nurses.

**Objective:**

The study aimed to analyze the international landscape of the nursing informatician role, comparatively focusing on the situation in Spain, to subsequently identify the specific gaps for its implementation through experts’ views and insights.

**Methods:**

First, a scoping review following the PRISMA-ScR (Preferred Reporting Items for Systematic Reviews and Meta-Analyses Extension for Scoping Reviews) guidelines was conducted in English and Spanish using scientific evidence searched in PubMed, Scopus, CINAHL, and Web of Science from 2018 to the present, as well as gray literature on the topic. A total of 55 published studies were included after screening 1356 records and 10 gray literature documents. Subsequently, findings were validated through a gap analysis comprising a panel of 10 experts selected according to their experience in digital literacy.

**Results:**

The review identified 6 core competencies for nursing informaticians: information management, cybersecurity, patient safety, evaluation and development of clinical information systems, leadership and coordination of digital tools, implementation of new technologies and specialized applications, and education and digitalization in health. Internationally, training is delivered via postgraduate programs, certifications, and leadership initiatives. Experts validated the relevance of these competencies for Spain (rated 5/5) and the applicability and desirability of implementing training programs (rated 4.8/5). Key barriers identified were the lack of official recognition, scarce training, and organizational resistance to change.

**Conclusions:**

There is a contrast between the established role of nursing informaticians internationally and its absence in Spain. The lack of a formal framework and specific training programs is the primary barrier. Implementing validated competencies and tailored educational strategies is crucial for Spain to advance its digital health transformation and empower nursing leadership in technology.

## Introduction

### Global Perspective

Nursing informatics (NI) is a discipline that has gained relevance in recent years due to the increasing integration of information and communication technologies. The definition of NI includes the integration of information technologies in patient care and the clinical setting. Digital transformation facilitates and enhances diagnosis, data management, information, and optimization of processes. This can lead to improved quality of care and efficiency of the health care system [1]. Within this transformation, NI emerges as a crucial discipline that integrates nursing science, information technology, innovative technologies, and data analytics to manage and communicate data, information, technology integration, and nursing practice [1,2]. The professionals who lead this charge are nursing informaticians (in Spanish “Enfermeras Especialistas en Sistemas de Información” or “Enfermeras Informáticas” [EESIs]), who serve as the vital connection between clinical practice, information systems, and technical teams [3].

### Literature Review

In countries like the United States, Canada, and Australia, this advanced role is well-established and robustly supported. The presence of professional bodies such as the American Medical Informatics Association, the Healthcare Information and Management Systems Society, the Canadian Nursing Informatics Association, and the Health Informatics Society of Australia provides a structured framework for professional development. These frameworks help define the role of nursing informaticians as a link between clinical practice and information systems that analyze, design, implement, and evaluate health information technologies. “Nursing informatician” is a generic term that includes variations depending on the formative level and leadership. For example, “nurse informaticist” or “nursing informatics specialist” is a common and specific job title for a professional with advanced knowledge [4]. Other alternatives are “chief nursing informatics officer,” an executive role that provides an informatics strategy focused on nursing practice, and “chief X informatics officer,” which includes clinical informatics leaders from various disciplines [5]. Furthermore, universities in these nations have prioritized the integration of informatics competencies into nursing curricula, empowering graduates to lead the use of technology in clinical practice [6]. This combination of professional communities, academic programs, and defined competency frameworks enables nursing informaticians to lead high-impact projects in the digital transformation of health care systems.

This leadership is present in several key areas. For instance, nursing informaticians can contribute to the development of interactive digital platforms to supervise and ensure the quality of clinical placements. In this context, a user-centered design has proven fundamental to successful adoption by supervisors and students [7,8]. Their expertise becomes essential in guiding institutions through the creation or development of e-learning platforms, where success depends on a broad and systematic analysis of needs rather than on technical specifications alone [9]. Moreover, they command the “co-creation” of educational resources, mostly virtual teaching packages. This methodology involves end users in the design process, creating training tools perceived as relevant and useful by them [10].

Emerging technologies, such as artificial intelligence, language processing models, big data, and 3D printing, can also be part of these nurses’ competencies. However, without appropriate training and a legislative framework, the integration of these advances into clinical practice can be challenging. Their training enables them to stay updated on technological advancements [11].

These examples of NI competencies that are commonly assumed in the international context by their role underscore a profound functional gap in Spain. There, the nursing informatician’s role is not formally recognized, and relevant university training is scarce. This absence raises a key question: “Why is the concept of nursing informaticians developed and implemented globally while absent in Spain?” As experts point out, digital health in the country faces significant challenges due to a lack of specialized training and a defined competency framework for nursing in this field [8]. This requires defining specific competencies that go beyond just using clinical information systems [12]. Furthermore, nursing students’ basic digital literacy in their current studies is insufficient [13], so specialized training becomes essential to prepare future nurses to use and integrate technology in health care [14].

### Research Objectives

Given the differences between international and national paradigms, a comprehensive analysis was required. This led to a 2-phase study design comprising 2 objectives: the first was to analyze the international scientific and gray literature on the nursing informaticians’ role, comparatively focusing on the situation in Spain. The second objective consisted of validating these findings by identifying the specific barriers and opportunities for implementing the role in Spain according to experts’ views and insights.

## Methods

In line with the objectives, the design was divided into 2 phases: the first was related to the main objective for the scoping review, and the second phase focused on the second aim related to a gap analysis.

### Phase 1

A scoping review was chosen to conduct a comprehensive analysis of existing literature, identify research gaps in publications, and examine the diverse evidence surrounding the nursing informaticians’ role, as well as gray literature. Analysis is required before drawing specific conclusions [15]. To guide this review, a research question was framed using the population, concept, context framework:

- Population: the nursing profession and, specifically, the role of nursing informaticians
- Concept: professional roles, competencies, training models, barriers, and facilitators for the implementation of the nursing informaticians’ role
- Context: the international health care landscape, considering experienced countries in NI, is used as a benchmark to analyze the existing gap in the Spanish health care system

The scoping review was conducted following the PRISMA-ScR (Preferred Reporting Items for Systematic Reviews and Meta-Analyses Extension for Scoping Reviews) guidelines (Checklist 1) [16]. A comprehensive search strategy was developed to reflect the international scope and the specific focus on Spain. Key terms in both languages, English and Spanish, included “nursing informatics,” “nursing informatics,” “nursing informaticians,” “informática aplicada a la enfermería,” “enfermeras especialistas en sistemas de información,” and “enfermería informática.” The search was performed for articles published from 2018 to the present across high-impact databases: PubMed, Scopus, CINAHL, and Web of Science. Filters for language, document type, and full-text accessibility were applied. To ensure a comprehensive overview, the search was supplemented with gray literature from international NI associations, relevant institutional documents, and university curricula in Spain related to the discipline.

### Inclusion and Exclusion Criteria

Articles selected during the methodological analysis process contained one or more of the keywords corresponding to NI and nursing informaticians. In the next step, the content of the screened articles was studied to determine their relevance to the current study.

During the screening process, the following exclusion criteria were applied to scientific articles:

- Nonspecific article content despite mentioning the search terms
- Low academic quality: studies with inconsistent methodology or insufficient bibliographic support
- Full text unavailable
- Article irretrievable from repositories

After screening, a review of all literature was conducted following the steps recommended by the Joanna Briggs Institute (Table 1) for conducting scoping reviews. This process included identifying the research question, searching for relevant evidence, selecting studies, extracting data, and presenting the results [15].

**Table 1.** Joanna Briggs Institute appraisal tools.

Main author [reference] and title	Article type	Critical appraisal tools	Item 1	Item 2	Item 3	Item 4	Item 5	Item 6	Item 7	Item 8	Item 9	Item 10	Item 11	Include
Reid L et al [1]. Defining nursing informatics: a narrative review.	Narrative Review	JBI^a^ Critical Appraisal Checklist for Textual Evidence: Narrative	1^b^	1	1	1	1	3^c^	3	1	3	1	1	1
Sensmeier J [2]. The value and impact of the alliance for nursing informatics.	Program Evaluation	JBI Critical Appraisal Checklist for Textual Evidence: Narrative	1	1	1	1	1	1	—^d^	—	—	—	—	1
Kirchner RB [3]. Presentando a la enfermera especialista en sistemas de información.	Narrative Article	JBI Critical Appraisal Checklist for Textual Evidence: Narrative	1	1	1	1	1	1	—	—	—	—	—	1
Benavente-Rubio A [6]. El rol de enfermería en la salud digital.	Systematic Review	JBI Critical Appraisal Checklist for Systematic Reviews and Research Syntheses	1	1	1	1	1	3	3	1	1	1	1	1
Konstantinidis ST [7]. HEALINT4ALL digital interactive platform for European and national placements audit for medicine and allied health professions following a user-centered design.	Book chapter	JBI Critical Appraisal Checklist for Textual Evidence: Narrative	1	1	1	1	1	1	—	—	—	—	—	1
Papamalis F [8]. Ensuring quality health care practice for doctors and medical allied professionals through a digital interactive audit platform.	Conference paper	JBI Critical Appraisal Checklist for Textual Evidence: Narrative	1	1	1	1	1	1	—	—	—	—	—	1
Baggia A [9]. Ecosystem of organizations in the digital age: Conference proceedings.	Conference paper	JBI Critical Appraisal Checklist for Textual Evidence: Narrative	1	1	1	1	1	1	—	—	—	—	—	1
Konstantinidis S [10]. Co-creation of a virtual interactive teaching package for auditors of health care placements–towards assurance of quality of health care traineeships.	Conference paper	JBI Critical Appraisal Checklist for Textual Evidence: Narrative	1	1	1	1	1	1	—	—	—	—	—	1
Ball MJ. et al [11]. The health informatics series: evolving with a new discipline.	Narrative Review	JBI Critical Appraisal Checklist for Textual Evidence: Narrative	1	1	1	1	1	1	—	—	—	—	—	1
León Molina J [12]. Papel de enfermería en el sistema de información hospitalario.	Narrative Article	JBI Critical Appraisal Checklist for Textual Evidence: Narrative	1	1	1	1	1	1	—	—	—	—	—	1
Chipps J et al [13]. Nursing informatics skills relevance and competence.	Interview-based descriptive study	JBI Critical Appraisal Checklist for Qualitative Research	1	1	1	1	3	3	1	1	—	—	—	1
Backonja U [14]. How to support the nursing informatics leadership pipeline.	Descriptive cross-sectional study	JBI Critical Appraisal Checklist for Analytical Cross Sectional Studies	1	1	1	1	3	3	1	1	—	—	—	1
Peters MDJ [15]. Updated methodological guidance for the conduct of scoping reviews.	Publication guideline	JBI Critical Appraisal Checklist for Textual Evidence: Narrative	1	1	1	1	1	1	1	1	—	—	—	1
Page MJ et al [16]. Declaración PRISMA 2020.	Publication guideline	JBI Critical Appraisal Checklist for Textual Evidence: Narrative	1	1	1	1	1	1	1	1	—	—	—	1
Luppa N and Suresh S [4]. Physician and nurse informatics collaboration.	White Paper	JBI Critical Appraisal Checklist for Textual Evidence: Policy	1	1	1	3	1	2^e^	1	—	—	—	—	1
Conte G et al [17]. Embracing digital and technological solutions in nursing.	Scoping Review	JBI Critical Appraisal Checklist for Systematic Reviews and Research Syntheses	1	1	1	1	1	1	1	1	3	1	1	1
Farzandipour M et al [18]. Designing a national model for assessment of nursing informatics competency.	Quantitative study	JBI Critical Appraisal Checklist for Analytical Cross Sectional Studies	1	1	1	1	3	3	1	1	—	—	—	1
Farokhzadian J et al [19]. Necessary prerequisites for evidence-based practice.	Cross-sectional study	JBI Critical Appraisal Checklist for Analytical Cross Sectional Studies	1	1	1	1	1	1	1	1	—	—	—	1
Kulju E et al [20]. Educational interventions and their effects on health care professionals’ digital competence development.	Narrative Review	JBI Critical Appraisal Checklist for Textual Evidence: Narrative	1	1	1	1	1	1	1	2	3	1	1	1
Saco P [21]. Informática aplicada a la enfermería.	University Subject Guide	JBI Critical Appraisal Checklist for Textual Evidence: Narrative	1	1	1	2	1	1	—	—	—	—	—	1
Nes AAG et al [22]. Technological literacy in nursing education.	Systematic Review	JBI Critical Appraisal Checklist for Systematic Reviews and Research Syntheses	1	1	1	1	1	1	1	1	3	1	1	1
Yildiz M et al [23]. Hemşirelerde tiger temelli hemşirelik bilişimi yetkinlikleri.	Descriptive-correlational study	JBI Critical Appraisal Checklist for Analytical Cross-Sectional Studies	1	1	1	1	3	2	1	1	—	—	—	1
Canadian Nurses Association [24]. Nursing informatics.	Policy Brief	JBI Critical Appraisal Checklist for Textual Evidence: Policy	1	1	1	3	1	2	1	—	—	—	—	1
Ramos Rodríguez JM [25]. Las TICs en enfermería de práctica avanzada.	Narrative Article	JBI Critical Appraisal Checklist for Textual Evidence: Narrative	1	1	1	1	1	1	—	—	—	—	—	1
González-Pardo Maza E [26]. Tecnología Big Data y su misión en el campo de la enfermería.	Narrative Review	JBI Critical Appraisal Checklist for Textual Evidence: Narrative	1	1	1	1	1	1	—	—	—	—	—	1
Reid L et al [27]. Nursing informatics: competency challenges for nursing faculty.	Scoping review	JBI Critical Appraisal Checklist for Systematic Reviews and Research Syntheses	1	1	1	1	1	1	1	1	3	1	1	1
AMIA^f^ Policy Invitational [28]. Redefining our picture of health.	White Paper	JBI Critical Appraisal Checklist for Textual Evidence: Policy	1	1	1	1	1	1	1	—	—	—	—	1
Toffoletto MC and Ahumada Tello JD [29]. Telenursing in care, education, and management.	Integrative Review	JBI Critical Appraisal Checklist for Systematic Reviews and Research Syntheses	1	1	1	1	1	3	3	1	3	1	1	1
Kaihlanen A et al [30]. Nursing informatics competence profiles and perceptions.	Cross-sectional study	JBI Critical Appraisal Checklist for Analytical Cross Sectional Studies	1	1	1	1	1	1	1	1	—	—	—	1
Forman TM et al [31]. A review of clinical informatics competencies in nursing.	Systematic Review	JBI Critical Appraisal Checklist for Systematic Reviews and Research Syntheses	1	1	1	1	1	3	3	1	3	1	1	1
Kleib M et al [32]. Approaches for defining and assessing nursing informatics competencies.	Scoping Review (protocol)	JBI Critical Appraisal Checklist for Systematic Reviews and Research Syntheses	1	1	1	1	1	3	1	2	2	2	1	1
Kleib M et al [33]. Approaches for defining and assessing nursing informatics competencies.	Scoping Review	JBI Critical Appraisal Checklist for Systematic Reviews and Research Syntheses	1	1	1	1	1	1	1	1	1	1	1	1
Davies A et al [34]. Core competencies for clinical informaticians.	Systematic Review	JBI Critical Appraisal Checklist for Systematic Reviews and Research Syntheses	1	1	1	1	1	1	1	1	1	1	1	1
Domingos CS et al [35]. A aplicação do processo de enfermagem informatizado.	Narrative Review	JBI Critical Appraisal Checklist for Textual Evidence: Narrative	1	1	1	1	1	1	—	—	—	—	—	1
Nahm ES et al [36]. Cybersecurity essentials for nursing informaticists.	Narrative Review	JBI Critical Appraisal Checklist for Textual Evidence: Narrative	1	1	1	1	1	3	1	3	3	1	1	1
Taylor-Pearson K et al [37]. The role of nurse informaticians in advancing 3D printing use in health care.	Systematic Review	JBI Critical Appraisal Checklist for Systematic Reviews and Research Syntheses	1	1	1	1	1	1	1	1	3	1	1	1
Australia’s Digital Health Community [5]. Leadership in clinical informatics.	White Paper	JBI Critical Appraisal Checklist for Textual Evidence: Policy	1	1	1	1	1	3	1	—	—	—	—	1
Brown J et al [38]. Issues affecting nurses’ capability to use digital technology.	Integrative Review	JBI Critical Appraisal Checklist for Systematic Reviews and Research Syntheses	1	1	1	1	1	1	1	1	1	1	1	1
Gonzalo de Diego B et al [39]. Competencies in the robotics of care for nursing robotics.	Scoping Review	JBI Critical Appraisal Checklist for Systematic Reviews and Research Syntheses	1	1	1	1	1	3	1	2	3	2	1	1
Nazeha N et al [40]. A digitally competent health workforce.	Scoping review	JBI Critical Appraisal Checklist for Systematic Reviews and Research Syntheses	1	1	1	1	1	1	1	1	1	1	1	1
Le Y et al [41]. A bibliometric and visualized analysis of nursing informatics competencies in China.	Bibliometric Review	JBI Critical Appraisal Checklist for Textual Evidence: Narrative	1	1	1	1	3	3	1	1	—	—	—	1
von Gerich H et al [42]. Artificial intelligence-based technologies in nursing.	Scoping review	JBI Critical Appraisal Checklist for Systematic Reviews and Research Syntheses	1	1	1	1	1	1	1	1	3	1	1	1
Harerimana A et al [43]. Nursing informatics in undergraduate nursing education.	Scoping Review	JBI Critical Appraisal Checklist for Systematic Reviews and Research Syntheses	1	1	1	1	1	3	1	1	1	1	1	1
Wynn M et al [44]. Digital nursing practice theory.	Scoping review	JBI Critical Appraisal Checklist for Systematic Reviews and Research Syntheses	1	1	1	1	1	3	1	1	3	1	1	1
Oh J et al [45]. Evaluation of the effects of flipped learning of a nursing informatics course.	Quasi-experimental study	JBI Critical Appraisal Checklist for Quasi-Experimental Studies	1	1	1	1	1	1	3	1	1	3	2	1
Lokmic-Tomkins Z et al [46]. Perspectives on the implementation of health informatics curricula frameworks.	Qualitative study	JBI Critical Appraisal Checklist for Qualitative Research	1	1	1	1	3	2	1	1	—	—	—	1
Haupeltshofer A et al [47]. Promoting health literacy.	Scoping review	JBI Critical Appraisal Checklist for Systematic Reviews and Research Syntheses	1	1	1	1	1	1	1	1	3	1	1	1
Lee KH et al [48]. Empowering healthcare through comprehensive informatics education.	Narrative Review	JBI Critical Appraisal Checklist for Textual Evidence: Narrative	1	1	1	1	1	3	3	1	3	1	1	1
Stunden A et al [49]. Nursing students’ preparedness for the digitalized clinical environment.	Integrative Review	JBI Critical Appraisal Checklist for Systematic Reviews and Research Syntheses	1	1	1	1	1	1	1	1	1	1	1	1

a JBI: Joanna Briggs Institute.

b 1: yes (valid).

c 3: No (not valid).

d Not applicable.

e 2: unclear.

f AMIA: American Medical Informatics Association.

### Data Extraction and Analysis

This review was conducted through manual data extraction by the researchers to maximize the quality of information selection. The search strategy was supplemented by reviewing gray literature, obtaining documents from relevant associations, and white papers on NI around the world. Some were deemed eligible, and others were excluded after a screening process. The data were then processed in an ordered format with the help of the Microsoft Office suite. This helped create a much more sensible and accurate starting point for the analysis using that software. This method likely ensures a high-quality review of the literature and, in turn, provides a rigorous summary of the evidence.

Artificial intelligence was used on a very limited basis to make only editorial corrections within the final manuscript in order to enhance writing quality and correct grammar, spelling, and structure in different article files. These tools were not used to extract or analyze results. This approach ensures the relevance and currency of the extracted data within the study context.

### Phase 2

This phase aimed to validate the scoping review findings through a gap analysis, consisting of a cross-sectional expert panel consultation survey with quantitative items and related qualitative open-ended questions (this survey or questionnaire can be found in Multimedia Appendix 1). For this purpose, a panel of 10 national experts in NI was assembled. All participants volunteered to participate and were recruited through an open call issued by the authors. Experts were selected based on the following criteria: (1) 10 or more years of experience related to health and digital literacy environments in Spain, (2) extensive training in digital literacy, and (3) peer-recognized expertise within their professional field. Their sociodemographic characteristics are detailed in Table 2.

**Table 2.** Experts’ sociodemographic characteristics.

ID	Gender	Age (y)	Years of experience	Highest education level	Current position	Main area of expertise	Autonomous community
E1	Male	46	22	PhD	University professor	Digital health literacy	Madrid
E2	Male	53	29	PhD	Director of Digital Strategy	Hospital information systems	Catalonia
E3	Female	49	25	Master’s	Clinical Informatics Specialist	Clinical workflows and EHR^a^	Andalusia
E4	Female	57	31	PhD	Senior eHealth Researcher	Telemedicine and patient care	Madrid
E5	Female	40	16	Master’s	Informatics Unit Supervisor	EHR implementation	Valencian Community
E6	Male	32	15	PhD	Digital Health Consultant	Data governance and security	Valencian Community
E7	Female	43	19	PhD	Health care Innovation Manager	Semantic interoperability	Galicia
E8	Female	56	33	PhD	Dean of Health Sciences	Ethics and legislation in eHealth	Castile and León
E9	Male	45	20	Master’s	Advanced Practice Nurse	Big data and health analytics	Valencian Community
E10	Female	53	27	PhD	Director of Nursing Services	Leadership and digital change	Andalusia

a EHR: electronic health record.

Sample size was determined based on the principles of purposive sampling and data saturation. This approach is consistent with the methodological literature on Delphi studies and expert panels, where a sample of 10 to 20 highly specialized participants is considered sufficient to achieve a comprehensive depth of information and reach thematic saturation [50]. Qualitative comments from the experts were analyzed through a simplified thematic analysis to identify relevant information not captured by quantitative items. The quality and richness of the data provided by these experts were prioritized over a larger, less specialized sample. To ensure objectivity, anonymity among experts was maintained so that none of the experts knew the identity of the rest of the panel.

### Ethical Considerations

The scoping review conducted in this study exclusively used publicly available data and literature. Therefore, it did not require ethical approval. For the gap analysis, all participating experts were informed of the study’s objectives and provided written consent to participate before answering the questionnaire. Anonymity and confidentiality of their responses were guaranteed throughout the process. No compensation was provided to the participants.

### Trial Registration

The results of a health care intervention on human subjects are not reported in this study; therefore, registration in a clinical trials registry was not applicable.

## Results

### Scientific Literature Review

**Figure 1.**
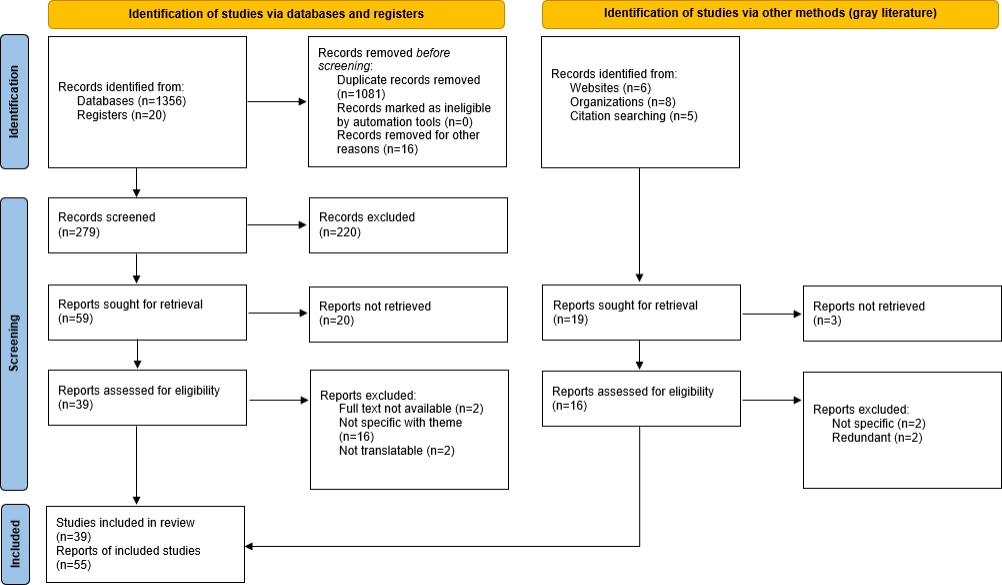
PRISMA (Preferred Reporting Items for Systematic Reviews and Meta-Analyses) flow diagram.

The scoping review included 55 published studies (the full list is available in Multimedia Appendix 2). The literature review identified an increasing scientific output internationally on NI and the role of nursing informaticians (see the PRISMA [Preferred Reporting Items for Systematic Reviews and Meta-Analyses] flow diagram [Figure 1] for the process of the complete search [16]). However, production in Spanish is significantly lower.

### Academic Training

During the study and after an exhaustive literature analysis, several training methods were identified that are used to impart the necessary knowledge and skills to nursing informaticians in clinical practice (Table 3).

**Table 3.** Academic training.

Academic training	Description	References
Undergraduate and postgraduate programs	Degrees incorporating health information systems, data analysis, and emerging technologies.	[1,17-19]
Research projects and theses	Advanced training (Master’s/PhD) focused on investigating specific NI^a^ problems to develop analytical skills.	[18]
Practical training and rotations	Supervised clinical placements allowing the application of theoretical knowledge in real-world settings.	[2,17,19]
Simulations and labs	Use of EHR^b^ simulators and informatics laboratories for safe, interactive learning.	[17,26,27]
Continuing education	Professional certifications, seminars, and workshops for updating skills (eg, cybersecurity, data management).	[2,20,23,24,29]
Leadership programs	Mentorship initiatives for emerging leaders (eg, Alliance for Nursing Informatics Emerging Leaders Program).	[2,25]

a NI: nursing informatics.

b EHR: electronic health record.

In summary, training in NI is conducted through a combination of academic programs, competency models, continuing education, clinical placements, simulations, online courses, seminars, workshops, research projects, and leadership training. These training methods must be developed to increase knowledge about NI [30] and evaluated to ensure that nurses acquire the necessary competencies to effectively implement and use information systems in clinical practice, as well as to integrate new technologies into the clinical environment [31].

### Competencies

As a result of the consulted literature and its exhaustive analysis, essential competencies for nursing informaticians have been identified [32,33]. These competencies are fundamental to understanding the functions they would fulfill in this nursing leadership role within the Spanish health care system. They are structured into 6 general competencies (Table 4).

**Table 4.** Competencies.

Competencies	Description	References
Information management	Includes the collection, storage, organization, and analysis of health data. Nursing informaticians must ensure patient privacy (applying current regulations) regarding their private information at all times. It also implies establishing standardized nursing documentation models for efficient and accurate information (NI^a^ process), as well as the use of standardized taxonomies. Measures nursing care complexity and ensures privacy.	[35,36,51,52]
Cybersecurity and patient safety	The integration of new technologies must prioritize patient safety, minimizing risks. In turn, the accuracy and reliability of data must be guaranteed, as well as their confidentiality.	[36,37]
Evaluation and development of clinical information systems	Analysis and management, creation, evaluation, and modification of hospital information systems to improve the quality of care. They must be practical and relevant to nursing practice. They must also integrate connectivity with new technologies.	[36]
Leadership and coordination of digital tools	Leadership in digital communication and patient care management using advanced digital tools are important competencies.	[5,14,36]
Implementation of new technologies and specialized applications	Integration of artificial intelligence, 3D printing, metaverse, big data, and robotics. Nursing informaticians as technological experts integrating and promoting these technologies.	[37-40]
Education and digitalization in health	Continuing education in new technologies is crucial to prepare health professionals. Nursing informaticians must be trained to transmit knowledge in information technologies and the use of emerging technologies to health care professionals in their environment.	[41,42]

a NI: nursing informatics.

### Contextual Framework

The successful implementation of nursing informaticians requires the definition, training, and aptitude of nurses in digital competencies within a specific contextual framework. This framework must include both generalist nurses and nursing informaticians [17,38,47].

All nurses must have the opportunity to receive training and qualifications as expert users to fully leverage the possibilities offered by technology while also knowing how to mitigate information overload [5,38]. Generally, younger nurses tend to possess more positive training, education, and attitudes toward technology; however, all professionals must be provided with opportunities adapted to their existing knowledge to undergo training in digital transformation [17,38]. Strategies must be established to combat computer illiteracy, structural problems (such as lack of staff and financial resources), and limited access to new technologies, which constitute the most common barriers. Likewise, these conditions must be met while ensuring the privacy and security of obtained data, servers, and devices to guarantee the safety of both patients and professionals [28,38].

Specific leadership roles in digital transformation will be assumed by nursing informaticians [5,17]. Their objective must be to increase system efficiency and improve the quality of care through evidence-based data in an interdisciplinary, coordinated, and patient-centered manner [4,5,17,24].

### Benefits

Based on the literature, the implementation of the nursing informaticians’ role in Spain presents several potential benefits, inspired by the experiences of other countries (Table 5).

**Table 5.** Benefits.

Benefits	Description	References
Improved management and use of health information systems	Nursing informaticians combine clinical nursing knowledge with an understanding of information sciences, acting as a connection between nurses and technical teams	[1,3,4,23,28,32,33,47]
Leadership in the adoption of new technologies	Responsible for developing and implementing ICTs^a^ in the clinical setting	[3,8,14,22,24,38,47]
Optimization of the development and implementation of the electronic health record	Nursing informaticians ensure that EHR^b^ systems are designed to facilitate compatibility with care and traceability of clinical practice	[3,18,30,38,42]
Contribution to nursing research	Participating in the research and implementation of digitalization solutions, as well as the collection, processing, and interpretation of large datasets	[6,14,15,28,29,40,46]
Effective application of the NP^c^, care complexity, and resource allocation	Strengthening evidence-based practice and consolidating nursing as a science. Nursing informaticians can ensure the effective application of the NP and promote the use of standardized taxonomies. Furthermore, the use of structured data allows quantifying care complexity. Consequently, nursing informaticians become essential for designing systems that move from operational documentation to predictive decision-making	[8,19,23,35,42,51-53]

a ICT: information and communication technology.

b EHR: electronic health record.

c NP: nursing process.

### Barriers

Potential barriers to its implementation have been identified (Table 6).

**Table 6.** Barriers.

Barriers	Description	References
Lack of formal recognition and specific training programs in Spain.	Currently, training in NI^a^ is limited.	[13,19,25,32,33,35,42,48]
Need to improve digital literacy in nursing.	Current competencies in applied informatics are insufficient.	[8,13,19,26,35,38,42]
Challenges in the integration of technologies and systems.	Includes a plurality of terminologies and a lack of experience among nurses.	[6,43,49]
Absence of a national digital health competency framework specific to nursing.	Essential for defining and acquiring the competencies of nursing informaticians.	[6,17,43]
Resistance to change and a lack of understanding of the value of structured data.	On the part of some professionals.	[17,26,42]
Limitations in accessing and using clinical nursing data.	Depend on electronic records.	[2,6,13,42,43]
Need for investment and resources.	For the training and integration of these professionals.	[5,6,14,20,38,46]

a NI: nursing informatics.

### Expert-Validated Gap Analysis

Following the scoping review, the opinions of a panel of 10 experts in the field of NI were gathered. Using a form, they evaluated the results retrieved from the scoping review based on their extensive experience. They rated items on a scale from 1 (strongly disagree) to 5 (strongly agree) and wrote down their perspectives on each aspect from the scoping review.

### Academic Training

The applicability and desirability of implementing specific training programs for nursing informaticians were overwhelmingly rated very positively by the experts (4.5/5). The proposals for their implementation are diverse:

- Continuing education: the importance of “continuing education from professional associations” is emphasized, including “practical workshops” and accessible courses. A “hybrid training model, in small teams, by knowledge level and very real needs applied to the specific practice of the work area” is proposed.
- Methodology: the development of a “modular and progressive curriculum, with training in digital competencies from the degree level, interdisciplinary master’s programs, and accredited continuing education, integrating mixed methodologies (in-person and online), digital clinical simulations, and practicums in real health care settings” is valued.
- Collaboration: the need for universities, in collaboration with professional associations and technology companies, to work in symbiosis to develop the most relevant content is emphasized.
- Implementation phases: some experts suggest “starting with pilot experiences in hospitals that are already digitized” or “initially through postgraduate training, possibly due to low demand,” to later integrate it into the undergraduate degree.
- Culture and recognition: the importance of “creating the culture and the social or professional symbolic image of this role before its future implementation” and the need for a “prior change in the culture of digital literacy among nursing professionals” are highlighted.

### Competencies

The competencies described in the scoping review received the highest rating (4.7/5), along with the benefits. The experts consider that the competencies identified for nursing informaticians are “highly relevant and applicable within the Spanish health care system, as they directly respond to current and emerging needs”:

- Key benefits: it is highlighted that they are crucial for “patient safety” and the “improvement in quality of care, potential for research, and analysis of results.”
- Adaptation to the environment: it is acknowledged that they must be “adapted to our reality: limited resources, differences between autonomous communities, and the need for more practical training.”
- Transdisciplinary role: it is suggested that nursing informaticians “should be integrated into transdisciplinary teams in research, as well as in management or decision-making regarding the design, development, validation, and implementation of new technologies.”
- European trend: it is pointed out that “they correlate with basic European digital competencies, therefore showing traceability with our educational system.”

### Benefits

There was broad recognition among experts (4.7/5) of the multiple benefits that implementing the EESI role would bring:

- Improved quality and efficiency: an “optimization in the management and use of health information systems” is expected, improving “care continuity, interoperability […] and the traceability of care.”
- Leadership in digital transformation: EESIs would provide “key leadership in the adoption and implementation of information and communication technologies.” One expert believes that “nursing is the most qualified profession to enhance its training in digital competencies and lead technological projects.”
- Boost to research: “nursing research would be promoted through the analysis of clinical data, allowing evidence to be generated from practice.”
- Strengthening the profession: the EESI role would help to “revalue the nursing profession as a key agent in the digital transformation of health.” The aim is for nurses to lead technological change, “(that we) do not just follow it.”
- Patient benefits: “care plans and health education, monitoring of care plans,” and the promotion of “active patient participation in their care process” are mentioned.

### Barriers

The experts’ ratings in this section were the most varied (4.4/5). Although the majority continue to identify significant obstacles, one expert is optimistic, noting that “all the classically identified barriers are being progressively addressed, which creates a more favorable and realistic scenario for the effective implementation of nursing informaticians’ “role,” while the others refer to these previously identified barriers:

- Lack of recognition and regulatory framework: the “absence of official recognition of the role” and the lack of a “regulatory framework and defined policies” are crucial.
- Training and digital literacy: the “scarce specific training at the national level (and a) low level of digital literacy in the nursing community” are mentioned. One expert indicates that “the main barrier is the nursing professionals themselves, due to a lack of time and a culture of digital competencies.”
- Resistance to change and organizational culture: “resistance to change from health care professionals accustomed to more traditional work models,” and the “lack of clinical leadership from nurses who are experts in information systems” are relevant.
- Resources and infrastructure: “scarce economic, technological, and human resources” and “difficulties in systems integration and terminological standardization” are impediments.

### Main Findings

This study reveals a significant dichotomy between the internationally recognized role of the nursing informaticians and their unrecognized role within the Spanish health care system. Through a scoping review, the international evidence has been synthesized, delineating a clear framework of competencies, training models, and benefits associated with this specialized role. Second, the expert-validated gap analysis confirmed that this framework is not only relevant because of its benefits but also essential for the Spanish context nowadays. Barriers that impede its implementation were also identified. The consensus among experts (4.7/5 for benefits and competencies) underscores a professional and academic mandate to bridge this gap.

Findings articulate 6 core competencies for the nursing informaticians: information management, cybersecurity and patient safety, evaluation and development of clinical information systems, leadership and coordination of digital tools, implementation of new technologies and specialized applications, and education and digitalization in health. These are more than technical skills, as they represent functions such as empowering nurses to act as the central professionals between clinical practice, information technology, and organizational strategy [3,29]. The strong validation by Spanish experts (4.7/5) confirms their applicability and designates a direction for future educational and professional development programs regarding this role.

## Discussion

### Comparison With Spanish Legal Framework

The status of NI in Spain has proven to be distanced from that of countries such as the United States, Canada, and Australia, where professional associations like the American Medical Informatics Association and the Healthcare Information and Management Systems Society have led the integration of nursing informaticians as those responsible for driving digital transformation [2,8,12,23,25]. A critical analysis of the Spanish legal framework reveals the structural nature of this gap. While international nursing informaticians possess advanced competencies in systems design, cybersecurity, and data leadership, the current regulation of the nursing profession in Spain—specifically Order CIN/2134/2008, which regulates the nursing degree [54], and Royal Decree 954/2015 on nursing prescription [55]—limits digital competencies to the user level.

Both regulations solely and explicitly define the digital competency of nurses in Spain as “applying technologies and information systems” [54,55]. This creates a dichotomy: the legislation exclusively restricts nurses to using existing tools, whereas the health care system increasingly demands qualified specialists to evaluate, design, and lead the implementation of new tools. This restriction within the legal framework illustrates why Spanish nursing staff often feel insufficiently prepared for the digitized clinical environment, echoing findings from Australia [13,49].

### Frameworks for Transformation and Cultural Change

A proposed conceptual framework as a driver for change could be the Technology Informatics Guiding Education Reform, with the intent of moving from basic computer literacy toward information management and innovation leadership. The progression of these competencies would reflect the Data-Information-Knowledge-Wisdom model, in which nursing informaticians in Spain would guide digital transformation by using health information and resources to create solutions adapted to the Spanish context.

Overcoming these barriers requires a deliberate and structured strategy, similar to that already achieved by other international pioneers. The emphasis placed by the experts consulted in this study on the “lack of a digital literacy culture” and “resistance from the professionals themselves” points to the need for a cultural shift. Technical training must be accompanied by an evolution of the organizational culture that values data-driven care and nursing leadership in technology.

### Implications for Practice, Policy, and Future Research

The findings of this study have significant implications: hospital and health care managers can use the validated competency framework from this study to formally introduce the role requirements. Providing nurses with specialized training and empowering them to lead improvement projects related to information systems or the implementation of new digital tools could potentially create momentum and demonstrate the value of the role so that they are not only technical but also strategic for making nursing care visible [17,26,42,51].

Results suggest a call to action for policymakers and regulatory bodies in Spain, such as the Ministry of Health and National Agency for Quality Assessment and Accreditation, to integrate nursing informaticians’ competencies into nursing curricula. This could begin with undergraduate introductions and extend to interdisciplinary master’s programs, as suggested by the experts. Collaboration among academia, professional associations, and technology companies is essential to ensure that this training is relevant, practical, and aligned with the needs of the health care system.

Longitudinal studies are needed to evaluate the impact of pilot training programs on nursing competencies. Further research should also focus on developing and validating a standardized instrument to assess NI competencies specifically tailored to the Spanish health care context.

### Limitations

This study has several limitations that should be acknowledged. First, the search was limited to studies published in English, Spanish, and Portuguese, which may have excluded relevant literature in other languages. The manuscript has been carefully reviewed by a native English speaker, although some phrasing or constructs may still reflect the linguistic influences of the researchers’ native language. Second, the search of gray literature may not have captured all relevant institutional documents or unpublished curricula. Third, the expert panel was selected through purposive sampling to ensure richness of data. Although thematic saturation was reached, the sample was limited to 10 participants. Furthermore, a potential selection bias exists due to the voluntary nature of the recruitment (open call); the experts may possess a baseline “pro-technology” attitude, which could have influenced the highly positive ratings compared to what might be observed in a generalist nursing population. Fourth, the protocol for this scoping review was not prospectively registered in a public repository such as PROSPERO (International Prospective Register of Systematic Reviews). However, the study was conducted following a detailed internal protocol developed by the authors following the PRISMA-ScR and Joanna Briggs Institute guidelines. Finally, a potential publication bias in the literature was spotted, where successful implementations of NI are more likely to be reported than failures. This could skew the international perspective. Future research should address the operational and budgetary requirements needed to change the organizational governance model for implementing the nursing informaticians’ role.

### Conclusions

This 2-phase study provides a comprehensive and critical diagnosis of the situation of NI and their role in Spain. It revealed a gap between the nation’s current reality and the established international standard. The lack of a conceptual framework, specialized university training, and official recognition for the nursing informaticians’ role are the principal barriers preventing nursing informaticians from leading the digital transformation of health care.

The international literature findings were validated by national experts, creating an actionable roadmap. The identified competencies offer a robust foundation for designing educational programs. The successful training models from other countries provide a blueprint for their implementation. The experts’ high level of agreement was unequivocal: the nursing informaticians’ role is imperative for improving patient safety, optimizing system efficiency, and furthering the development of NI as a scientific discipline in Spain.

Challenges are significant, although the pathway forward is clear. This research serves as a foundational call to action for policymakers, academic institutions, and health care leaders: building regulatory, educational, and organizational infrastructure is needed to empower a new generation of nursing leaders equipped to lead the future of digital health in Spain.

## Supplementary material

10.2196/83373 Multimedia Appendix 1 Gap analysis questionnaire.

10.2196/83373 Multimedia Appendix 2 Scoping review chart 1.

10.2196/83373 Checklist 1 PRISMA checklist.

## Abbreviations

EESI: Enfermeras Especialistas en Sistemas de Información
NI: nursing informatics
PRISMA: Preferred Reporting Items for Systematic Reviews and Meta-Analyses
PRISMA-ScR: Preferred Reporting Items for Systematic Reviews and Meta-Analyses Extension for Scoping Reviews
PROSPERO: International Prospective Register of Systematic Reviews
