# Correction: Nursing Informaticians in Spain: Scoping Review and Expert-Validated Gap Analysis

**DOI:** 10.2196/100481

**Published:** 2026-08-11

**Authors:** Adrián Marco-Moyano, Patricia Verdú Rodríguez, Manuel Lillo-Crespo

**Affiliations:** 1 University of Alicante, San Vicente del Raspeig, Alicante, Spain; 2 Faculty of Health Sciences, University of Alicante, Carretera de San Vicente del Raspeig, s/n, San Vicente del Raspeig, Alicante, 03690, Spain, 34 965903400 ext 3518

In “Nursing Informaticians in Spain: Scoping Review and Expert-Validated Gap Analysis” [1], the authors noted one error.

The corresponding author information has been changed from:

Adrián Marco-Moyano, RN, BSN, MSN

Nursing, Faculty of Health Sciences, University of Alicante

Carr. de San Vicente del Raspeig, s/n 965 90 34 00

San Vicent del Raspeig, Alicante 03690

Spain

Phone: 34 681281259

Email: adrian.marco@grupohla.com

It now appears as follows:

Manuel Lillo-Crespo, RN, BSN, MBAExec, MSN, MSCAnthro, PhD

Faculty of Health Sciences, University of Alicante

Carretera de San Vicente del Raspeig, s/n

San Vicente del Raspeig, Alicante 03690

Spain

Phone: 34 965903400 ext 3518

Email: manuel.lillo@ua.es

This information was added to the affiliations list as affiliation 2.

In addition, affiliation 1 was changed from:

Nursing, Faculty of Health Sciences, University of Alicante, San Vicent del Raspeig, Alicante, Spain

It now appears as:

University of Alicante, San Vicent del Raspeig, Alicante, Spain

The correction will appear in the online version of the paper on the JMIR Publications website together with the publication of this correction notice. Because this was made after submission to PubMed, PubMed Central, and other full-text repositories, the corrected article has also been resubmitted to those repositories.
