# Supplementary material for: Nursing Informaticians in Spain: Scoping Review and Expert-Validated Gap Analysis
Source: JMIR Nurs. 2026 Apr 27;9:e83373. doi: 10.2196/83373 (PMC13119383; doi:10.2196/83373)
Supplement: Multimedia Appendix 1 [file nursing-v9-e83373-s001.pdf]

Questionnaire

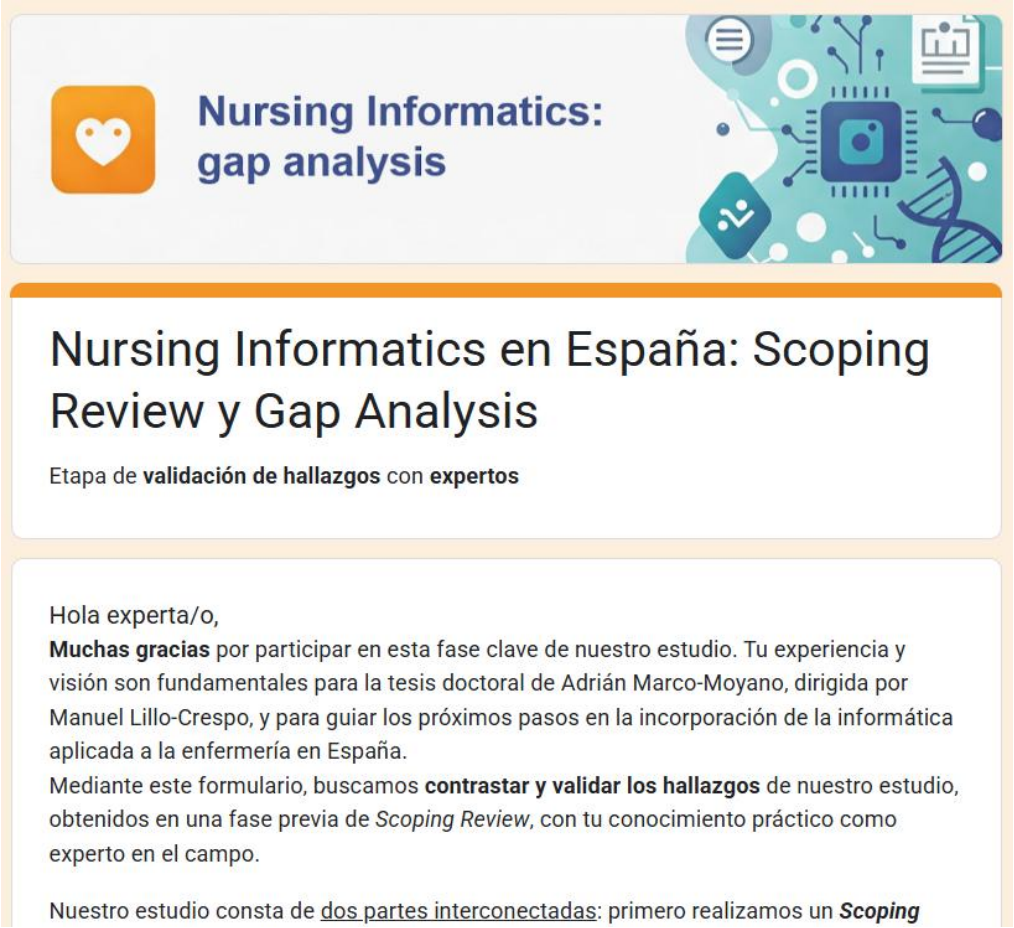
Nursing Informatics: gap analysis

Nursing Informatics en España: Scoping Review y Gap Analysis

Etapa de **validación de hallazgos** con **expertos**

Hola experta/o,
Muchas gracias por participar en esta fase clave de nuestro estudio. Tu experiencia y visión son fundamentales para la tesis doctoral de Adrián Marco-Moyano, dirigida por Manuel Lillo-Crespo, y para guiar los próximos pasos en la incorporación de la informática aplicada a la enfermería en España.
Mediante este formulario, buscamos **contrastar y validar los hallazgos** de nuestro estudio, obtenidos en una fase previa de *Scoping Review*, con tu conocimiento práctico como experto en el campo.

Nuestro estudio consta de dos partes interconectadas: primero realizamos un ***Scoping***

Original Questionnaire picture

Translated copy of the questionnaire

Page 1

Nursing Informatics in Spain: Scoping Review and Gap Analysis

Hello expert,
Thank you very much for participating in this key phase of our study. Your experience and vision are fundamental for the doctoral thesis of Adrián Marco-Moyano, supervised by Manuel Lillo-Crespo, and for guiding the next steps in the incorporation of informatics applied to nursing in Spain.

Through this form, we seek to contrast and validate the findings of our study, obtained in a previous Scoping Review phase, with your practical knowledge as an expert in the field.

Our study consists of two interconnected parts: first, we conducted a Scoping Review analyzing the international literature on the competencies of Information Systems Specialist Nurses (EESI), training models, and challenges for their implementation in Spain.

In this Gap Analysis phase, we seek to validate these results with you.

To facilitate your response, the form is organized into sections. Initially, we will provide you with a summary of the study's context, then the key results of our review, and finally, you will find the validation questions. Your task is to read the results section (section 3) and answer the questions in the last section (section 4) based on your expert judgment to share your unique perspective.

Your answers will be treated as strictly confidential. From the beginning, this questionnaire is designed so that you cannot be linked to your answers, and they will be used solely for the purposes of this research.

https://docs.google.com/forms/d/1LJMEIJZOfVW-1ZuHz2AcQmnR9_ffBgy1T0FghmElqUA/edit

1/9

Page 2

Context

Nursing Informatics is a key discipline focused on integrating ICTs to improve patient care and efficiency. Information Systems Specialist Nurses (EESI), called NIs or Informatics Nurses in other countries, act as a bridge between technology, patients, and healthcare staff, always prioritizing quality of care and safety.

In countries like the United States, Canada, or Australia, this role is already consolidated, with specific training and associations that define competencies and promote technological leadership in care. However, in Spain, the EESI role does not yet have formal recognition, and university education in this area is very scarce.

https://docs.google.com/forms/d/1LJMEIJZOfVW-1ZuHz2AcQmnR9_ffBgy1T0FghmElqUA/edit

2/9

Page 3

Results

The international scientific literature shows a growing interest in nursing informatics. However, academic production in Spanish is considerably lower, which highlights the need to analyze and adapt the experiences and models of other countries to the Spanish context.

Academic Training

The training of EESI at the international level is multifaceted (theory, practice, research, leadership) and requires continuous evaluation and improvement to ensure that nurses acquire the necessary competencies:

1. Formal academic programs: universities around the world offer specific undergraduate and postgraduate degrees with an interdisciplinary focus (informatics, nursing, management). The importance of applied informatics and digital literacy is highlighted, often through mixed approaches (face-to-face and e-learning). In Spain, the offer is scarce.
2. Research: at the master's and doctoral levels, research in nursing informatics is promoted, developing analytical skills.
3. Competency models: there are frameworks (e.g., Digitech-F, TIGER...) to define, evaluate, and guide the development of digital competencies in nursing.
4. Diversified practical training: includes continuing education, certifications, leadership programs (e.g., Emerging Leaders Program), clinical placements, simulations, online courses, and workshops on specific topics (e.g., cybersecurity).

Competencies

The analysis of the literature identifies six key competency areas for EESI, essential for their leadership role:

1. Information management: encompasses the collection, storage, organization, and analysis of health data, ensuring privacy and using standards and taxonomies.
2. Cybersecurity and patient safety: involves integrating technology with a priority on patient safety, minimizing risks, and ensuring data integrity and confidentiality.
3. Evaluation and development of clinical information systems: consists of analyzing, managing, evaluating, and adapting information systems to optimize the quality of care.
4. Leadership and coordination of digital tools: leading the effective use of digital

communication and managing patient care with advanced technological tools.
5. Implementation of new technologies and specialized applications: includes the active integration and promotion of emerging technologies such as AI, 3D printing, big data, and robotics in clinical practice.
6. Education and digitalization in health: consists of training other health professionals in ICTs and emerging technologies, promoting continuous training and digital literacy.

https://docs.google.com/forms/d/1LJMEIJZOfVW-1ZuHz2AcQmnR9_ffBgy1T0FghmElqUA/edit

3/9

Page 4

Benefits

The introduction of the EESI role in Spain could bring significant advantages based on international experiences:

- Optimization in the management and use of health information systems, acting as a link between clinic and technology.
- Leadership in the adoption and implementation of ICTs.
- Improvement in the development and use of the electronic health record.
- Promotion of nursing research through data analysis.
- Strengthening of the application of the Nursing Process (NP) in digital format and the use of standardized languages.

Barriers

The implementation of this role in Spain faces significant obstacles:

- Absence of official recognition and specific training programs at the national level.
- Need to improve the general digital literacy of nurses.
- Difficulties in the integration of heterogeneous systems and lack of terminological standardization.
- Lack of a national framework of digital health competencies for nursing.
- Possible resistance to change among professionals.
- Limitations in the access and use of structured clinical data.
- Requirement of investment in training and resources to integrate these specialists.

Rate your degree of agreement with these results (each section), below:

https://docs.google.com/forms/d/1LJMEIJZOfVW-1ZuHz2AcQmnR9_ffBgy1T0FghmElqUA/edit

4/9

Page 5

Academic training:

1 = Totally disagree

2 = Disagree

3 = Neither agree nor disagree (Neutral)

4 = Agree

5 = Totally agree

Competencies:

1 = Totally disagree

2 = Disagree

3 = Neither agree nor disagree (Neutral)

4 = Agree

5 = Totally agree

Benefits:

1 = Totally disagree

2 = Disagree

3 = Neither agree nor disagree (Neutral)

4 = Agree

5 = Totally agree
https://docs.google.com/forms/d/1LJMEIJZOfVW-1ZuHz2AcQmnR9_ffBgy1T0FghmElqUA/edit

5/9

Page 6

<u>Barriers</u>:

Nursing Informatics in Spain: Scoping Review and Gap Analysis

1 = Totally disagree

2 = Disagree

3 = Neither agree nor disagree (Neutral)

4 = Agree

5 = Totally agree

1 2 3 4 5

Expert's Vision

Now that you have reviewed the context of the study and the results of our Scoping Review, we invite you to share your expert perspective. Your vision is crucial to validate, refine, and contrast these findings; to understand their applicability and viability in the Spanish healthcare system. Please answer the following questions based on your knowledge and experience. Feel free to elaborate as much as you consider necessary.

1. Considering the training models explored in the literature, how do you rate the feasibility of implementing specific training programs for EESI in Spain from your perspective as a professional in the sector? How would you carry it out?
    i. Results: multifaceted training composed of formal academic programs, research, competency models, and diversified practical training.

https://docs.google.com/forms/d/1LJMEIJZOfVW-1ZuHz2AcQmnR9_ffBgy1T0FghmElqUA/edit

6/9

Page 7

2. Based on the EESI competencies identified in the international literature, what

is your expert opinion on their relevance and applicability within the Spanish healthcare system?

i. Results: information management, cybersecurity and patient safety, evaluation and development of clinical information systems, leadership and coordination of digital tools, implementation of new technologies and specialized applications, and education and digitalization in health.

3. From your expert point of view, what do you identify as potential benefits of the effective implementation of the EESI role in Spain?
   i. Results: optimization in the management and use of health information systems, acting as a link between clinic and technology, leadership in the adoption and implementation of ICTs, improvement in the development and use of the electronic health record, promotion of nursing research through data analysis, strengthening of the application of the Nursing Process (NP) in digital format and the use of standardized languages.

https://docs.google.com/forms/d/1LJMEIJZOfVW-1ZuHz2AcQmnR9_ffBgy1T0FghmElqUA/edit

7/9

Page 8

4. From your perspective, what would you consider barriers to the effective implementation of the EESI role in Spain?
   i. Results: absence of official recognition and specific training programs at the national level, need to improve the general digital literacy of nurses, difficulties in the integration of heterogeneous systems and lack of terminological standardization, non-existence of a national framework of digital health competencies for nursing, possible resistance to change among professionals, limitations in the access and use of structured clinical data, requirement of investment in training and resources to integrate these specialists.

Thanks for your participation!

Again, thank you very much for your time and for sharing your valuable experience. Your contribution is fundamental to our research. We will keep you informed about the final publication of the study.

This content is not created or endorsed by Google.

https://docs.google.com/forms/d/1LJMEIJZOfVW-1ZuHz2AcQmnR9_ffBgy1T0FghmElqUA/edit

8/9

Page 9

Nursing Informatics in Spain: Scoping Review and Gap Analysis

https://docs.google.com/forms/d/1LJMEIJZOfVW-1ZuHz2AcQmnR9_ffBgy1T0FghmElqUA/edit

9/9
