# Supplementary material for: Nursing Informaticians in Spain: Scoping Review and Expert-Validated Gap Analysis
Source: JMIR Nurs. 2026 Apr 27;9:e83373. doi: 10.2196/83373 (PMC13119383; doi:10.2196/83373)
Supplement: Multimedia Appendix 2 [file nursing-v9-e83373-s002.docx]

Table 1 - JBI Appraisal tools

Table 2 – Experts’ Sociodemographic Characteristics

| **ID** | **Gender** | **Age (Years)** | **Years of exp.** | **Highest education level** | **Current position** | **Main area of expertise** | **Autonomous Community** |
| --- | --- | --- | --- | --- | --- | --- | --- |
| **E1** | Male | 46 | 22 | PhD | University professor | Digital Health Literacy | Madrid |
| **E2** | Male | 53 | 29 | PhD | Director of Digital Strategy | Hospital Information Systems | Catalonia |
| **E3** | Female | 49 | 25 | Master's | Clinical Informatics Specialist | Clinical Workflows & EHR | Andalusia |
| **E4** | Female | 57 | 31 | PhD | Senior eHealth Researcher | Telemedicine & Patient Care | Madrid |
| **E5** | Female | 40 | 16 | Master's | Informatics Unit Supervisor | EHR implementation | Valencian Community |
| **E6** | Male | 32 | 15 | PhD | Digital Health Consultant | Data governance & security | Valencian Community |
| **E7** | Female | 43 | 19 | PhD | Healthcare Innovation Manager | Semantic interoperability | Galicia |
| **E8** | Female | 56 | 33 | PhD | Dean of Health Sciences | Ethics & legislation in eHealth | Castile and León |
| **E9** | Male | 45 | 20 | Master's | Advanced Practice Nurse | Big data & health analytics | Valencian Community |
| **E10** | Female | 53 | 27 | PhD | Director of Nursing Services | Leadership & digital change | Andalusia |

Table 3 - Academic training

| **Academic Training** | **Description** | **References** |
| --- | --- | --- |
| Undergraduate and postgraduate programs | Degrees incorporating health information systems, data analysis, and emerging technologies. | [1, 18, 24, 25] |
| Research projects and Theses | Advanced training (Master/PhD) focused on investigating specific NI problems to develop analytical skills. | [24] |
| Practical training and rotations | Supervised clinical placements allowing application of theoretical knowledge in real-world settings. | [2, 18, 25] |
| Simulations and labs | Use of EHR simulators and informatics laboratories for safe, interactive learning. | [18, 31, 32] |
| Continuing Education (CNE) | Professional certifications, seminars, and workshops for updating skills (e.g., cybersecurity, data management). | [2, 21, 26, 29, 33] |
| Leadership programs | Mentorship initiatives for emerging leaders (e.g., ANI Emerging Leaders Program). | [2, 30] |

Table 4 - Competencies

| **Competencies** | **Description** | **References** |
| --- | --- | --- |
| Information management | Includes the collection, storage, organization and analysis of health data. NIs must ensure patient privacy (applying current regulations) regarding their private information at all times. It also implies establishing standardized nursing documentation models for efficient and accurate information (NI process), as well as the use of standardized taxonomies. Measure nursing care complexity and ensure privacy. | [38, 39, 51, 53] |
| Cybersecurity and patient safety | The integration of new technologies must prioritize patient safety, minimizing risks. In turn, the accuracy and reliability of data must be guaranteed, as well as its confidentiality. | [39, 40] |
| Evaluation and development of clinical information systems | Analysis and management, creation, evaluation and modification of hospital information systems to improve the quality of care. They must be practical and relevant to nursing practice. They must also integrate connectivity with new technologies. | [39] |
| Leadership and coordination of digital tools | Leadership in digital communication and patient care management using advanced digital tools are important competencies. | [5, 14, 39] |
| Implementation of new technologies and specialized applications | Integration of artificial intelligence, 3D printing, metaverse, big data and robotics. NIs as technological experts integrating and promotioning these technologies. | [22, 40 – 42] |
| Education and digitalization in health | Continuing education in new technologies is crucial to prepare health professionals. NIs must be trained to transmit knowledge in information technologies and the use of emerging technologies to healthcare professionals in their environment. | [43, 44] |

Table 5 – Benefits

| **Benefits** | **Description** | **References** |
| --- | --- | --- |
| Improved management and use of health information systems | NIs combine clinical nursing knowledge with an understanding of information sciences, acting as a connection between nurses and technical teams | [1, 3, 4, 20, 23, 29, 36, 37] |
| Leadership in the adoption of new technologies | Responsible for developing and implementing ICTs in the clinical setting | [3, 8, 14, 20-22, 28] |
| Optimization of the development and implementation of the electronic health record | Optimization of the development and implementation of the electronic health record | [3, 22, 24, 34, 44] |
| Contribution to nursing research | participating in the research and implementation of digitalization solutions, as well as the collection, processing and interpretation of large datasets | [6, 14, 15, 23, 33, 42, 48] |
| Effective application of the Nursing Process (NP), care complexity and resource allocation | strengthening evidence-based practice and consolidating nursing as a science. NIs can ensure the effective application of the NP and promote the use of standardized taxonomies. Furthermore, the use of structured data allows quantifying care complexity. Consequently, NIs become essential for designing systems that move from operational documentation to predictive decision-making | [8, 25, 29, 38, 44, 51-53] |

Table 6 – Barriers

| **Barriers** | **Description** | **References** |
| --- | --- | --- |
| Lack of formal recognition and specific training programs in Spain. | Currently, training in NI is limited. | [13, 25, 30, 36-38, 44, 49] |
| Need to improve digital literacy in nursing. | Current competencies in applied informatics are insufficient. | [8, 13, 22, 25, 31, 38, 44] |
| Challenges in the integration of technologies and systems. | Includes a plurality of terminologies and a lack of experience among nurses. | [6, 45, 50] |
| Absence of a national digital health competency framework specific to nursing. | Essential for defining and acquiring the competencies of NIs. | [6, 18, 45] |
| Resistance to change and a lack of understanding of the value of structured data. | On the part of some professionals. | [18, 31, 44] |
| Limitations in accessing and using clinical nursing data. | Depend on electronic records. | [2, 6, 13, 44, 45] |
| Need for investment and resources. | For the training and integration of these professionals. | [5, 6, 14, 22, 26, 48] |
